# Innovative Clinical Perspectives for CIK Cells in Cancer Patients

**DOI:** 10.3390/ijms19020358

**Published:** 2018-01-25

**Authors:** Martino Introna, Fabio Correnti

**Affiliations:** USS Center of Cell Therapy “G. Lanzani”, USC Ematologia, ASST Papa Giovanni XXIII Bergamo, 24124 Bergamo, Italy; fabio.correnti@gmail.com

**Keywords:** CIK, NK, GvHD

## Abstract

Cytokine-induced killer (CIK) cells are T lymphocytes that have acquired, in vitro, following extensive manipulation by Interferon gamma (IFN-γ), OKT3 and Interleukin 2 (IL-2) addition, the expression of several Natural Killer (NK) cell-surface markers. CIK cells have a dual “nature”, due to the presence of functional TCR as well as NK molecules, even if the antitumoral activity can be traced back only to the NK-like structures (DNAM-1, NKG2D, NKp30 and CD56). In addition to antineoplastic activity in vitro and in several in-vivo models, CIK cells show very limited, if any, GvHD toxicity as well as a strong intratumoral homing. For all such reasons, CIK cells have been proposed and tested in many clinical trials in cancer patients both in autologous and allogeneic combinations, up to haploidentical mismatching. Indeed, genetic modification of CIK cells as well as the possibility of combining them with specific monoclonal antibodies will further expand the possibility of their clinical utilization.

## 1. Introduction

Cytokine-induced killer (CIK) cells are non-MHC (Major Histocompatibility Complex) restricted, cytotoxic antitumoral cells expanded in vitro from circulating precursors. CIK cells share characteristics of both T and NK cells. Based on the published results obtained both in vitro and in vivo and with cells of both mouse and human origin, CIK cells show, in vivo, a very strong cytolytic activity against leukemia and graft versus leukemia (GVL), while being essentially devoid of graft-versus-host reactivity (GvHD).

Indeed, it has long been known that cytotoxic cells with this double T/NK phenotype are rare but present (from 1% to 5%) in circulating blood as originally described by Lanier [1], and are capable of lysing a broad array of tumor cell targets in a non-MHC-restricted manner.

### 1.1. CIK Cell Production: Cell Factory and Protocol

CIK cells are produced in vitro according to a specific expansion protocol originally discovered more than twenty years ago [2,3].

More recently, according to recent European and Italian laws (2003/94/EC), we have adapted this protocol to produce CIK cells for clinical experimental studies, strictly following the Good Manufacturing Practice (GMP) rules for drug products in our academic cell factory authorized by Agenzia Italiana del Farmaco (AIFA) (no. aM 57/2016). 

The cell factory is indeed listed in both the European EudraGMP (Available online: http://eudragmdp.ema.europa.eu/inspections/mia/searchMIA.do) and the AIFA institutional site (Available online: http://www.aifa.gov.it/sites/default/files/elenco_aziende_autorizzate_terapie_avanzate_30.09.2016.pdf) 

The protocol was approved by Istituto Superiore di Sanità (ISS) for the phase I study, 64499(04)-PRE21-848 and subsequent amendment 21862(07)-PRE21-848. 

The expansion protocol for the generation of CIK cells, starting from peripheral blood mononuclear cells (PBMNC), lymphocytoapheresis or cord blood requires the sequential addition of 1000 U/mL human rIFN-γ on day 0 followed by 50 ng/mL monoclonal antibody against CD3 OKT3 and 500 IU/mL rIL-2 on day +1. rIL-2 and fresh complete X-VIVO medium are added every five days as detailed in the specific SOP for 14–21 days.

### 1.2. CIK Cell Cultures Heterogeneity

In our own experience, we have determined that, at the end of the expansion procedure, two main cellular populations are present in the product: one cell type, usually referred to as CIK, which is CD3^−^, CD8^−^, NKG2D^−^ and CD56 positive; and a second population, which is CD3^−^, CD8^−^ and NKG2D positive but CD56 negative. Our studies have now clarified that: (1) the CIK cells are terminally differentiated nondividing cells which manifest the described nonspecific natural cytotoxicity; (2) the other population represents a progenitor reservoir that undergoes cell division and that differentiates into CIK cells, and this population is devoid of natural cytotoxic activity. It is still a matter of speculation whether the “maintenance” in vivo of the cytotoxic activity of the total cell culture is dependent on the presence of this proliferating reservoir of precursors; (3) the CIK cells are T cells that have acquired the natural cytotoxic potential of NK cells [4].

### 1.3. Mechanism of Action: Cytotoxic Activity of CIK Cells

CIK cells are endowed with a potent MHC-unrestricted cytotoxicity against both hematological and solid malignancies, and recognize and kill tumor targets without prior exposure or priming. The antitumor activity of CIK cells is mainly restricted to the CD3^+^ CD56^+^ fraction. Investigation into the possible explanations for the better cytolytic activities against tumor cells demonstrated for the CD3^+^ CD56^+^ cells over their CD3^+^ CD56^−^ counterpart, originally revealed that the CD3^+^ CD56^+^ cells consist of a higher proportion of CD8^+^ cells, as well as a more differentiated effector phenotype as well as a higher content of granzyme [5].

CIK cells were originally produced starting from peripheral blood-circulating mononuclear cells of normal donors (PB-CIK) and, subsequently, they could also be generated from mononuclear cells obtained from patients with newly diagnosed leukemia [6,7].

The CIK cells generated following culture were free from contamination of the original leukemic cells. It was demonstrated that CIK cells derived from leukemic patients with various chromosomal abnormalities such as t(15;17), t(8;21), Philadelphia chromosome, trisomies and complex structural abnormalities at diagnosis were totally free of all these chromosomal abnormalities on screening by karyotyping [7].

Similarly, Hoyle reported that CIK cells could be expanded also from patients with chronic myeloid leukemia (CML) [8]. In this case, CIK cells were shown to be cytolytic against autologous and allogeneic CML cells. Furthermore, CML colony growth was reported to be suppressed by CIK cells and after 28 days of co-incubation, the remaining colonies in culture were exclusively composed of Philadelphia-negative cells [8,9,10].

Furthermore, CIK cells could be generated from untreated chronic lymphocytic leukemia (CLL) patients and were cytotoxic against autologous CLL targets [11,12].

In the allogeneic combination, CIK cells showed cytotoxicity at the same levels as in the autologous combination against myeloid targets, but lysed poorly lymphoid blasts [7].

A comparative gene expression analysis of CIK cells in response to acute myeloid leukemia (AML) or acute lymphoblastic leukemia (ALL) stimulators revealed a differential regulation of immune-related genes [13].

Ex-vivo expanded human CD3^+^ CD56^+^ T cells produce cytokines of the T helper 1 (TH1) type and have broad non-MHC-restricted cytotoxicity against a variety of tumor cell lines as well as autologous and allogeneic fresh tumor isolates [3]. As a few examples, CIK cells have shown cytotoxic activity in vitro against alveolar rhabdomyosarcoma, ewing sarcoma, glioblastoma multiforme and retinoblastoma cell lines, amongst others [14,15,16].

### 1.4. Mechanism of Action: Receptors Involved in Activity of CIK Cells

CIK cells demonstrate cytolytic activity superior to lymphokine-activated killer (LAK) cells without the requirement of rhIL-2 treatment in vivo. Stimulation with IFN-γ results in IL-12 production from monocytes as well as upregulation of CD2 that interacts with leukocyte function-associated antigen 3 (LFA-3) for optimal in-vitro expansion of the human CD3^+^ CD56^+^ effector cells [17].

The mechanisms underlying the cytotoxicity of CIK cells have not yet been completely clarified, however some key molecules and pathways have been identified. Tests with blocking antibodies against CD2, CD3, CD8, CD28, CD56, very late antigen 4 (VLA-4), T-cell receptor (TCR) αβ, and MHC class I and II molecules failed to inhibit the cytotoxic activity, suggesting an MHC-independent method of target recognition. A significant inhibition was obtained blocking lymphocyte function-associated antigen 1 (LFA-1) and intracellular cell adhesion molecule 1 (ICAM-1), suggesting that cytolysis is dependent on cell-to-cell contact [3,4,18]. Treatment of CIK cells with dibutyryl (db)-cAMP, which prevents the conversion of LFA-1 into a high-affinity receptor for ICAM-1, inhibited perforin and granzyme release of CIK cells triggered by both anti-CD3 monoclonal antibody (mAb) or tumor targets [19]. The use of immunosuppressive drugs like cyclosporine and FK506 prevented degranulation of CIK cells induced by CD3–TCR stimulation, but could not block the cytotoxicity triggered by the interaction with tumor targets [19].

The molecule that seems to play the most important role in tumor recognition by CIK cells is probably the natural killer group 2 member D (NKG2D) receptor. NKG2D is a member of the c-type lectin-activating receptor family that is evolutionarily conserved and is located within the NK gene complex on human chromosome 12p12-p13 [20]. NKG2D is expressed on all NK cells and is a promiscuous receptor that recognizes at least six counterligands. These include the MHC-class I-like molecules, MHC class I polypeptide-related sequence A (MICA) and MHC class I polypeptide-related sequence B (MICB), and members of the UL16 binding protein (ULBP) family (ULPB1-4), named for the ability of some members to bind to the UL-16 protein of cytomegalovirus. Interestingly, the ligands for NKG2D appear to have a pattern of expression that is relatively restricted to malignant tissue [21,22].

NKG2D expression is upregulated in CIK cells activated and expanded in vitro, and it is not restricted to the CD3^+^ CD56^+^ subpopulation. Studies with antibodies blocking the NKG2D molecules, small interfering RNA experiments and redirected cytolysis indicated that the majority of the cytotoxicity of CIK cells is exerted through the NKG2D interaction rather than TCR engagement [23,24]. The action of NKG2D is probably associated with the upregulation of the NKG2D-associated adaptor molecule disulphide adaptor protein 10 (DAP-10), involved in the NKG2D activatory signalling, induced by the high dose of rhIL-2 present in the culture medium of CIK cells [23]. More recently, NKG2D has been involved also in the target recognition by the IL-15 (instead of IL-2) in-vitro-expanded CIK cells, and reported to be more cytotoxic in vitro [19,25].

In-vitro data suggested that CIK cells were more effective against myeloid rather than lymphoid acute leukemia targets and that NKG2C and NKG2E as well as perforin were upregulated in the CIK cells only after the contact with myeloid targets. By contrast, the contact with lymphoid targets induced the upregulation of transforming growth factor beta (TGFb) in CIK cells [13].

Our previous studies clarified that CD3+CD56+ CIK cells have phenotypic characteristics typical of terminally differentiated CD8^+^ effector memory T cells (TEMRA; CCR7^−^, CD45RA^+^, CD62Llow, CD11a^+^, CD27^+^, CD28^−^). We also showed that CIK cells originate in vitro from CD56^−^CD8^+^ T cell progenitors which strongly expand upon culture in the presence of IL-2 and acquire CD56 antigen [4]. In addition, CIK cells share several characteristics with NK cells, such as the large granular lymphocyte morphology, the capacity to kill the HLA (Human leukocyte antigen)-class I-negative cell line K562, and the surface expression of high densities of CD56 and NKG2D. Different from NK cells, however, CIK cells express low densities of NKp30, whereas they do not express NKp44 and NKp46, and the inhibitory killer immunoglobulin-like receptors, NKG2A and CD94 [4].

We provided evidence that also DNAM-1 and NKp30 play a role in CIK cell-mediated antitumoral cytotoxicity. We showed that NKp30, although expressed at relatively low density, is involved in the recognition and killing of lymphoma targets [26]. Notably, a functional role of NKp30 in T cells has been described so far only in the case of IL-15 long-term cultured cord blood lymphocytes [27]. The expression of NKp30 in CIK cells is intriguing also in light of the reciprocal activation between CIK and dendritic cells [16,28,29,30] and of the crucial role of NKp30 in the interaction between NK cells and dendritic cells [31,32,33].

In the same avenue, it has been shown that CIK cells can kill immature dendritic cells (DC) by TCR-independent and perforin-dependent mechanisms [34]. 

**Figure 1:**
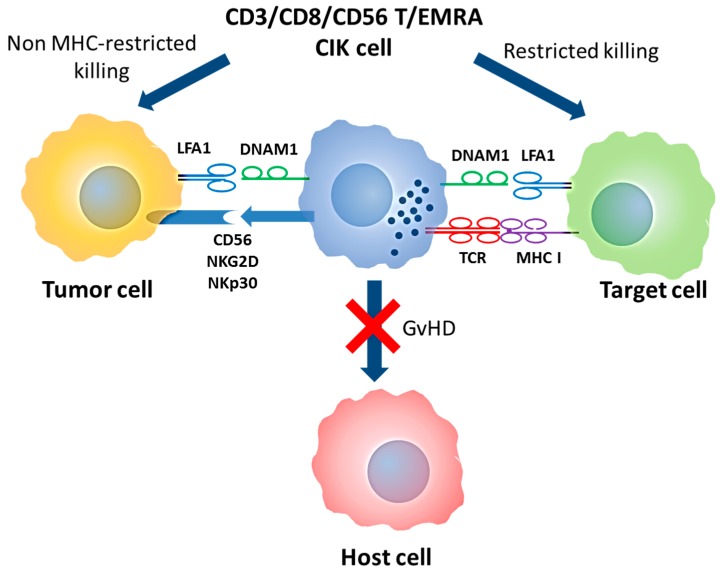
Double T/NK specificity of CIK cells and mechanism of action. CIK cells are T-EMRA lymphocytes that maintain the original TCR specificity and acquire NK-like cytotoxicity. In both cases, DNAM1 and LFA1 are required for target binding while NK-like cytotoxicity is mediated by CD56, NKp30 and NKG2D molecules. Abbreviation: MHC (Major Histocompatibility Complex); GVHD (Graft versus Host Disease); T/NK (T Natural Killer); CIK (Cytokine Induced Killer); T-EMRA (Effector memory T); TCR (T cell receptor); NK (Natural Killer); DNAM1 (DNAX Accessory Molecule-1); LFA1 (Lymphocyte function-associated antigen 1)

More recently, we also provided evidence that CD56 has a direct role in CIK-mediated cytotoxicity, at least against CD56 positive hematopoietic targets, suggesting that a homophilic interaction between CD56 molecules may occur in tumor cell recognition [35] (Figure 1).

### 1.5. Antitumoral Activity of Human CIK Cells In Vitro

Ex-vivo expanded human CD3^+^CD56^+^ CIK cells produce cytokines of the TH1 type and have broad non-MHC-restricted cytotoxicity against a variety of tumor cell lines as well as autologous and allogeneic fresh tumor isolates [3,6,7,8,12,17,23,36].

Importantly, human CIK cells did not show strong cytotoxic potential versus normal hematopoietic precursors. In one example, CFU-GM activity of human bone marrow cells was only partially impaired (75% of control) [37].

### 1.6. Antitumoral and Absence of GvHD Activity by Mouse CIK Cells in Mouse Models

CD8^+^ murine CIK cells do not lyse normal bone marrow (BM) or normal spleen cells in vitro (in colony-forming unit granulocyte macrophage assays) and they do not interfere with BM engraftment in vivo. Indeed, no toxicity was observed following the intravenous injection of up to 50 × 10^6^ CD8^+^ CIK cells [24].

In another study, expanded CD8^+^ CIK cells from C57BL/6 (H2b) mice displayed minimal to no cytotoxicity against autologous BM, but mediated potent antitumor activity against BCL-1 lymphoma tumor targets [38]. In the allogeneic situation, expanded CD8^+^ CIK cells from C57BL/6 (H-2b) mice readily killed both BM and tumor targets from Balb/c (H2d) animals. In all cases, the killing of the BM was reduced compared to that of the tumor targets, suggesting specificity for malignant cells [21].

More importantly, doses between 5 × 10^6^ and 20 × 10^6^ CD8^+^ CIK cells together with BM produced far less graft-versus-host disease (GVHD) than splenocytes across major histocompatibility (naive or CIK splenocytes from C57BL6 into irradiated Balb/c). Although these animals did experience mild weight loss as compared to animals that were transplanted with BM alone, all of the animals survived for reasons which at the moment are unclear, but are related to the production of IFN-γ by CIK cells, in addition to other Th1 cytokines such as tumor necrosis factor (TNF), as suggested by a subsequent paper by the same group of scientists [39] Indeed, CIK expanded from IFN-γ knock-out mice caused acute lethal GVHD [39]. Unexpectedly, the CIK cells were well tolerated even at doses significantly greater than the doses of unfractionated splenocytes or purified splenic CD8 cells which caused, in the same genotype combination, lethal disease [38].

Similar data have been obtained in different animal models: Hematopoietic stem cells (HSCs) and expanded CD8^+^ CIK cells from C57BL/6, as well as control splenocyte mice, were introduced into lethally irradiated MHC-mismatched Balb/c recipients together with Balb/c BCL-1 lymphoma. Mice that received naive splenocytes rapidly died within the first two weeks due to acute GVHD. In contrast, mice that received HSC plus expanded CD8^+^ CIK cells regained weight showing GVL activity without GvHD. Similar results were observed when the opposite strain combination was performed (Balb/c CIK into C57BL6 recipients) as well as across minor histocompatibility barriers (B10.d2 into Balb/c) [40].

In another interesting model, CIK generated from FVB mice were injected into allogeneic Balb/c with Balb/c BM cells. Control splenocytes killed all animals by GvHD, while mice receiving up to four times the same dose of CIK cells did not show any sign of GvHD. Interestingly, CIK homed to and proliferated in the spleen and cervical lymph nodes and expanded dramatically (luciferase-expressing cells) similarly to what was observed of splenocytes. However, the gut infiltration by CIK was slower and waxed and waned completely as well as in the small and large intestine. By contrast, CIK cells accumulated and persisted at tumor sites resulting in tumor eradication. A possible role of slower division rate of CIK cells and different patterns of expression of homing molecules are investigated [41].

### 1.7. Antitumoral Activity of Human CIK In Vivo in Animal Models

The first observations have been made in the C.B-17 SCID (severe combined immunodeficiency) model. Irradiated mice received bone marrow cells contaminated with human B lymphoma SU-DHL-4 cell line either in the presence or absence of CIK cells. Control animals all died within 40 days, while 80% receiving CIK remained alive. Interestingly, in the same model, Lymphokine Activated Killer (LAK) cells were ineffective [37]. In a subsequent study, SCID mice were inoculated with SU-DHL-4 cells and one day later with CIK or LAK cells. CIK-treated animals had significantly prolonged survival compared with control animals (median survival 90 days versus 58) or animals treated with LAK. 30% of the SCID mice became long-term survivors compared with none of the LAK-treated animals [2].

In a more recent work, CIK cells were expanded from several Chronic Myelogenous Leukemia (CML) patients and injected four weeks after inoculum of the autologous CML CD34^+^ blasts in Matrigel. In those mice that received CIK, half had no microscopic evidence of disease and in the remainder the sizes of the tumors were significantly smaller than the control mice [8].

Finally, using bioluminescence to monitor growth and regression of labelled human cervical carcinoma HELA cells in SCID mice, human CIK cells showed a strong activity with clear dose-response effects. The lower cell dosage of 1 × 10^6^ resulted in transient responses in 3/5 animals, whereas at 1 × 10^7^, half of the animals had complete resolution of tumor signals [42].

### 1.8. Studies in Patients

Recently, a detailed first report of the International Registry on CIK Cells (Available online: www.cik-info.org), which has registered up to 1800 patients at the moment of this writing, was published. It is interesting to show how many clinical trials have been already conducted in the world with CIK cells in cancer patients. In a recent review, the authors identified 11 clinical trials involving 426 treated patients. In 10/11 studies, autologous CIK cells were used. The total number of CIK cells injected ranged from 21.9 × 10^7^ to 5.2 × 10^10^. The number of CIK cells used per infusion ranged from 7.2 × 10^6^ to 2.1 × 10^10^. Patients were treated with up to 40 infusions [43].

It is important to underline that side effects of these treatments were minor with less than 10 cases reporting hematochemical variations. Mild hypotension, fever and chill, headaches, nausea and vomiting have been occasionally reported. 

From the point of view of the efficacy of the 384 (90.1%) patients where a response was reported, 24 (5.6%) had a complete response (CR), 27 (6.3%) had a partial response (PR), and 40 (9.4%) had a minor response. 161 (37.8%) patients had stable disease and 129 (30.3%) progressed [43]. The extreme heterogeneity of these studies, nonetheless, should be underlined and makes difficult a correct and full understanding of their real clinical efficacy.

### 1.9. CIK in the Autologous Setting

The first clinical report on the administration of autologous CIK cells for hematopoietic neoplasms was a phase I study for the treatment of relapsed lymphomas after autologous stem cell transplantation (HSCT). Nine patients, seven with advanced Hodgkin’s disease (HD) and two with non-Hodgkin’s lymphomas (NHL) received from one to three doses of cells ranging from 1 × 10^9^ to 10 × 10^9^ CIK cells/infusion, with CD56^+^ CIK cells representing 8–58% (median 22%) of the total cell population in the final product. Toxicity was minimal, as one patient developed mild hypotension, and a second, low-grade fever. Two patients had a partial response and two patients had stabilization of disease, one for more than 18 months [44].

A subsequent phase I study included six patients with advanced lymphomas (3 HD) and the schedule consisted of three cycles of cells at an interval of three weeks. The median number of infused cells per patient was 28 × 10^9^ (range 6–61) and the absolute number of CIK cells infused was 7 × 10^9^ (range 2.2–21 × 10^9^).

Only one patient experienced low-grade fever as toxicity. One patient with centroblastic NHL was still alive in complete response (CR) 44 months after administration. All other five patients did not show any clinical response to therapy [45].

Negative results have been reported on 13 acute myelogenous leukemia (AML) patients in remission post autologous peripheral blood HSCT and on 11 chronic myeloid leukemia (CML) patients on imatinib with PCR-positive minimal residual disease (MRD). These patients had received autologous CIK, but survival and relapse did not change in the AML group with respect to controls, and bcr–abl transcript did not change in the CIK-treated CML patients [46]. Also in this study, autologous CIK showed no toxicity, but in this case there was no evidence of antitumoral activity.

By contrast, a study investigating the administration in 20 elderly leukemic patients (more than 70 years old), with diverse hematological malignancies, of autologous CIK (2–3 × 10^9^) followed by subcutaneous injection of rhIL-2 (1 mU/day for 10 consecutive days repeated every four weeks) showed promising results. The patients were nine lymphomas, five myelodysplastic syndrome (MDS), two multiple myeloma (MM), three chronic lymphocytic leukemia (CLL) and one AML. Fourteen patients received eight cycles and six patients received four cycles of therapy. In addition to a dramatic effect on cancer-related symptoms and quality of life, the authors refer their observation of 11 CR (three were in progression before CIK therapy, five stable disease (SD) and one PR, two were already in CR) and additional seven PR (five were in PD, two were already in PR). Mean survival time was 20 months. There was no report of toxicity and the mean Karnofsky score was increased from 57% to 83% [47].

Subsequently, the same group reported the results of a smaller group of nine elderly patients (mean age 83 years), with diffuse large B cell lymphoma (DLBCL), who received eight cycles of CIK, as detailed above. No adverse reaction has been reported. Interestingly, all patients achieved a CR at the study endpoint and survival time ranged from 24 to 35 months [48].

Very recently, one report has suggested the possibility of expanding CIK cells for autologous use also from the blood of recurrent or refractory AML patients with high peripheral leukemia cell burden, but clinical results are too preliminary to be critically evaluated [49].

### 1.10. CIK in the Allogeneic Setting

Due to the very strong non-HLA-restricted NK-like cytotoxicity of CIK cells and, more importantly, on the basis of the preclinical observations that CIK almost completely lacks GVHD activity in an allogeneic setting, we, and subsequently others, have suggested that donor-derived CIK cells can be administered to lymphoma/leukemia patients who relapse after allo-HSCT.

Major clinical studies are summarized in Table 1.

The first published phase I study with allogeneic CIK cells included eleven patients with AML (*n* = 4), HD (*n* = 3), chronic myelomonocytic leukemia (CMML, *n* = 1), pre-B acute lymphoblastic leukemia (ALL, *n* = 1) and MDS (*n* = 2), all of whom had relapsed after sibling (*n* = 6) or matched unrelated donor (*n* = 5). The median number of CIK infusions was two (range 1–7) and the median number of total CIK cells was 12.4 × 10^6^/kg (range 7.2–87.4). Infusions were well tolerated and no acute or late infusion-related reactions were recorded. Acute GvHD of grade I and II was observed in four patients 30 days after the last CIK infusion and these progressed into extensive chronic GvHD in two cases. Disease progression and death occurred in six patients. One patient had stable disease, one had a hematological improvement and three achieved CR [50].

The study, as indicated by DM 2/03/2004 (Italian law), has been registered in the ISS database of gene and cell therapy (ISS No. 83) (Available online: https://www.iss.it/site/attivita/terapiaGC/).

Similarly, 18 patients with hematological malignancies received allogeneic CIK cells, following relapse after allogeneic HSCT (with matched sibling in all cases). CIK cells were given at escalating doses of 1 × 10^7^/kg, (*n* = 4), 5 × 10^7^/kg (*n* = 6) and 1 × 10^8^/kg (*n* = 8). Acute GvHD grades I–II was seen in two patients and one had limited chronic GvHD. After a median follow up of 220 months (range 1–69), the median overall survival time was 28 months and the median event-free survival was four months. All deaths were due to leukemia relapse [51].

In a more recent experience, 24 patients with hematological malignancies who relapsed after allogeneic HSCT (15 from sibling, nine from unrelated donors) were enrolled to receive allogeneic CIK cells. Only 20 patients had a donor available and 14 were actually infused. They received from three to 22 CIK infusions. Interestingly, two patients without a donor could also be treated, by the infusion of autologous peripheral blood-derived CIK cells, and these received eight and three infusions, respectively. Out of 16 treated patients, no response was seen in six of them. Five additional patients fell into the “unable to assess” group, due to the concomitant use of other agents that could have induced a response. Finally, for five patients, there was evidence suggestive of antitumor activity of CIK cells (these included two ALL patients, two HD patients and one AML patient). Interestingly, two of the responders had a response sustained for more than two years. Acute GvHD occurred in three patients and was in all cases easily treatable [52].

On the basis of the results of our phase I study, at the end of 2009, we started a phase II protocol authorized by the national authority (Agenzia Italiana del Farmaco, AIFA, Rome, Italy) and the local ethical committees (EC). 

This phase II multicenter study was authorized by Istituto Superiore di Sanità (ISS), and as for Advanced Therapeutic Medicinal Product (ATMP) regulations, approved by AIFA. The trial was registered (EUDRACT no. 2008-003185-26, ClinicalTrial.gov: NCT01186809).

Our study is an open-labeled, multicenter, exploratory phase IIA study to evaluate the safety (dose-finding) and efficacy of a sequential administration of donor-derived unmanipulated lymphocytes (DLI) followed by in-vitro expanded CIK cells, to patients with hematologic malignancies relapsing after related or unrelated allogeneic HSCT. Two infusions of unmanipulated Donor Lymphocyte Infusion (DLI) (1 × 10^6^/kg) were given with a minimum interval of three weeks. Three infusions of donor CIK cells were then administered according to a dose-escalating program, starting three weeks after the second DLI. CIK administrations were separated by three-week intervals. Up to four combinations of dose-escalating levels were provided in sequential order until the maximal tolerated dose (MTD) was reached. Indeed, the first triplet of patients was planned to receive CIK cells at the doses of 1 × 10^6^/kg, 1 × 10^6^/kg and 5 × 10^6^/kg, the second 1 × 10^6^/kg, 5 × 10^6^/kg and 5 × 10^6^/kg, the third 1 × 10^6^/kg, 5 × 10^6^/kg and 10 × 10^6^/kg, and the last triplet 5 × 10^6^/kg, 5 × 10^6^/kg and 10 × 10^6^/kg. In case of grade-II or more severe acute GvHD, the next scheduled infusion was to be suspended. Only grade-IV acute GvHD was considered the dose-limiting toxicity (DLT). Once we have identified the MTD, this same dose will be administered up to 40 patients in a two-stage Simon’s design.

Seventy-four patients who relapsed after allogeneic stem cell transplantation were enrolled in a phase IIA study and treated with the sequential infusion of donor lymphocytes (DLI) followed by cytokine-induced killer (CIK) cells. Seventy-three patients were available for the intention-to-treat analysis. At least one infusion of CIK cells was given to 59 patients, while 43 patients received the complete cell therapy planned (58%). Overall, 12 patients (16%) developed acute GvHD (aGvHD, of grade I–II in seven cases and grade III–IV in five). In eight of 12 cases, aGvHD developed during DLI treatment leading to interruption of the cellular program in three patients, while in the remaining five cases, aGvHD was controlled by steroid treatment thus allowing the subsequent planned administration of CIK cells. Chronic GvHD (cGvHD) was observed in 11 patients (15%). A complete response was observed in 19 (26%), partial response in three (4%), stable disease in eight (11%), and progression of disease in the remaining 43 patients (59%). At one and three years, the progression-free survival was 31% and 29%, while the overall survival (OS) was 51% and 40%, respectively. By multivariate analysis, the type of relapse, the presence of cGvHD and a short (<6 months) time from allo-HSCT to relapse were the significant predictors of survival. In conclusion, a low incidence of GvHD is observed after the sequential administration of DLI and CIK cells, and disease control can be achieved mostly after a cytogenetic or molecular relapse [53].

The study, as indicated by DM 2/03/2004 (Italian law), has been registered in the ISS database of gene and cell therapy (ISS no. 107) (Available online: https://www.iss.it/site/attivita/terapiaGC/).

All in all, the above mentioned studies demonstrate that peripheral blood CIK cells, both in autologous and in allogeneic combinations (matched), have demonstrated a weak but measurable clinical antileukemic activity, perhaps more pronounced in the case of old patients with minimal residual disease. The different source (between matched unrelated and matched sibling donors) did not show significant difference in terms of antitumoral activity. 

### 1.11. CIK in the Haploidentical Setting

CIK cells derived from genetically haploidentical donors have also been administered to patients and major clinical studies are summarized in Table 2.

Solid tumors for total of 303 patients with six different types of solid cancers were treated with CIK cells [54]. 133 received autologous CIK while 170 received allogeneic, haploidentical CIK cells. The cells were infused daily (more than 1 × 10^9^). A cycle of treatment comprised between three and five infusions and more than 5 × 10^9^ cells were infused per cycle. The total number of cells in the autologous group was between 1.76 × 10^10^ and 1.35 × 10^11^ (mean 2.11 ± 0.32 × 10^10^), and between 1.16 × 10^10^ and 6.71 × 10^10^ (mean 2.29 ± 0.36 × 10^10^) in the allogeneic group. 

Predominant adverse events included insomnia, fever, nausea and vomiting in nine (6.8%), eight (6.0%), two (1.5%) and one (0.8%), respectively, in the autologous group; and 11 (6.5%), 10 (5.9%), one (0.6%) and one (0.6%), respectively, in the haploidentical group. Physical improvement was observed in 21 (15.8%) and 28 (16.5%) and pain relief in 15 (11.3%) and 20 (11.8%) in the autologous and allogeneic groups, respectively.

Inside the same paper, there is a much more detailed analysis of the subgroup of 54 patients with non-small-cell lung cancers, 27 of whom were treated by allogeneic (haploidentical) CIK cells and 27 as controls with supportive therapy only. The median overall survivor (OS) was 11 months (8.6–13.4 months) and eight months (5.3–10.7) for patients receiving allogeneic versus supportive therapy, respectively. 

In the hemato-oncological field, less clinical data are available for the haploidentical CIK administration.

In a different report [55], after bortezomib treatment, two MM patients received haploidentical-derived CIK cells at escalating doses of 1 × 10^7^ CD3/kg, 5 × 10^7^ CD3/kg and 1 × 10^8^ CD3/kg. One patient achieved CR and remission lasted 12 months, while two patients achieved a very good partial response (VGPR). The administrations were accompanied by some malaise and occasional self-limited fever. No patient developed any sign of GvHD. 

In a very recent clinical study, four patients with relapses of hematological or solid malignancies received CIK infusions from original haploidentical donors. In total, 22 infusions (range 1–9 infusions per patient) were administered with escalating cell numbers ranging between 0.1 × 10^6^ to 98.2 × 10^6^ CD3^+^CD56^+^ cells/kg and a minimum interval of three weeks between infusions. After two to 11 months of follow up, the authors conclude that CIK cell infusions were well tolerated and that no acute GvHD was observed. Analyses showed clearance of blasts in leukemia patients. One patient with a solid tumor remained progression-free from five months, whereas the other progressed after two months [57]. As clearly documented in these reports, mismatched CIK cells have never been associated with severe toxicity at all doses administered, and only mild symptoms have been reported, from nausea to low-grade fever, possibly as a consequence of cytokine release in vivo. The update of this study indicates that 13 patients received haploidentical CIK therapy following molecular (10) or overt relapse (three). Overall, three developed grade-III aGvHD, whereas continued complete molecular remission was sustained in 9/13, and the remaining four experienced delayed hematologic relapse, further demonstrating the feasibility, safety and efficacy of haploidentical CIK treatment [56].

## 2. Conclusions

CIK cells can be easily expanded under strict GMP conditions, so as to allow the execution of clinical experimental studies.

In the onco-hematological field, CIK cells showed promising results, with relatively modest antileukemia activity, slightly superior to the unmanipulated DLI, but with a very interestingly low GvHD toxicity. 

In the immediate clinical perspective, CIK cells may be offered to treat minimal neoplastic disease, in consideration of their demonstrated antitumoral activity in vivo and in vitro, their intratumoral homing and, finally, their very reduced graft-versus-host activity. Indeed, CIK cells from either autologous or allogeneic donors (up to haploidentical combinations) have already been extensively used in both the hematologic and solid tumor arenas. We can envision future clinical studies with CIK cells against minimal tumor diseases, perhaps with innovative schedules of administration, such as higher and more frequent dosages and, more importantly, in combinations with other drugs already available in the clinic today in order to improve their in-vivo activity.

For example, our ancillary study with bispecific antibodies may show one possible pathway for the combination with soluble molecules [58]. Similarly, new monoclonal antibodies utilized to harness the immune reactivity of infiltrating T cells could be certainly studied in combination with possible immunotherapy including CIK, NK and others.

The antitumoral activity of CIK cells has been mainly traced back to an innate NK-like nonspecific cytotoxicity. Indeed, CIK cells, as we and others have demonstrated [26,59], possess a functional T cell receptor, but whether this structure is used for the antitumoral activity in vivo is not clear. Indeed, to implement CIK-mediated antitumoral activity, CIK cells have been transduced by exogenous and artificial TCR (CAR), as exemplified by the CD19-transduced CIK cells [60]. Therefore, it is also possible to imagine future clinical perspectives by analogous CAR transductions in CIK cell therapy platforms. 

Furthermore, CIK cells have been isolated and clinically tested also when derived from cord blood units. Also in this case, we can envision alternative usage of cord blood-derived CIK cells which could be easily identified for allo/haplo mismatching, due to the abundance of cord blood units stored in the banks with little utilization for clinical transplantation.

Finally, it is entirely possible to imagine a future usage of DLI- or cord blood-derived CIK cells (with various degrees of matching) even after CAR genetic modification, for the treatment of many tumors, either alone or in combination with specific antibodies.

## Figures and Tables

**Table 1:** Most relevant clinical studies with allogeneic CIK cells.

# Patients	Median Cells Infused	Acute Toxicity	GVHD	Reference
11	12.4 × 10^9^/kg	-	4 aGvHD I + II	[50]
			2 cGvHD	
18	from 1 × 10^7^/kg to 1 × 10^8^/kg	-	2 aGvHD I + II 1 cGvHD mild	[51]
24	-	-	3 aGvHD I	[52]
74	20 × 10^6^/kg	-	12 aGvHD (4 grade I; 3 grade II; 4 grade III; 1 grade IV)	[53]
			11 cGvHD (4 mild; 5 moderate; 2 severe)	

Abbreviation: CIK (Cytokine Induced Killer); GvHD (Graft versus Host Disease); aGvHD (acute GvHD); cGvHD (chronic GvHD).

**Table 2:** Most relevant clinical studies with haploidentical CIK cells.

^#^ Patients	Median Cells Infused	Acute Toxicity	GvHD	Reference
170 (solid cancers)	3–5 administrations × 5 × 10^9^	11 insomnia; 10 fever; 1 nausea; 1 vomiting	-	[54]
2	from 1 × 10^7^/kg to 1 × 10^8^/kg	-	-	[55]
13	from 0.1 × 10^6^/kg to 100 × 10^6^/kg	-	3 aGvHD III	[56]
